# Expression and significance of HMGB1, TLR4 and NF-κB p65 in human epidermal tumors

**DOI:** 10.1186/1471-2407-13-311

**Published:** 2013-06-26

**Authors:** Hui Weng, Yunhua Deng, Yuyan Xie, Hongbo Liu, Feili Gong

**Affiliations:** 1 Department of Immunology, Tongji Medical College, Huazhong University of Science and Technology, 13 Hangkong Road, Wuhan 430030, China; 2 Department of Dermatology, Tongji Hospital, Tongji Medical College, Huazhong University of Science and Technology, Wuhan 430030, China; 3 Department of Pathology, The 5th Affiliated Hospital of Sun Yat-Sen University, Zhuhai 519000, China

**Keywords:** HMGB1, TLR4, NF-κB, Seborrheic keratosis, Precancerous lesions, Squamous cell carcinoma

## Abstract

**Background:**

High mobility group protein box 1 (HMGB1) is a DNA binding protein located in nucleus. It is released into extracellular fluid where it acts as a novel proinflammatory cytokine which interacts with Toll like receptor 4 (TLR4) to activate nuclear factor-κB (NF-κB). This sequence of events is involved in tumor growth and progression. However, the effects of HMGB1, TLR4 and NF-κB on epidermal tumors remain unclear.

**Methods:**

Human epidermal tumor specimens were obtained from 96 patients. Immunohistochemistry was used to detect expression of HMGB1, TLR4 and NF-κB p65 in human epidermal tumor and normal skin specimens. Western blot analysis was used to detect the expression of NF-κB p65 in epithelial cell nuclei in human epidermal tumor and normal tissues.

**Results:**

Immunohistochemistry and western blot analysis indicated a progressive but statistically significant increase in p65 expression in epithelial nuclei in benign seborrheic keratosis (SK), precancerous lesions (PCL), low malignancy basal cell carcinoma (BCC) and high malignancy squamous cell carcinoma (SCC) (P <0.01). The level of extracellular HMGB1 in SK was significantly higher than in normal skin (NS) (P <0.01), and was higher than in SCC but without statistical significance. The level of TLR4 on epithelial membranes of SCC cells was significantly higher than in SK, PCL, BCC and NS (P <0.01). There was a significant positive correlation between p65 expression in the epithelial nuclei and TLR4 expression on the epithelial cell membranes (r = 0.3212, P <0.01).

**Conclusions:**

These findings indicate that inflammation is intensified in parallel with increasing malignancy. They also indicate that the TLR4 signaling pathway, rather than HMGB1, may be the principal mediator of inflammation in high-grade malignant epidermal tumors. Combined detection of p65 in the epithelial nuclei and TLR4 on the epithelial membranes may assist the accurate diagnosis of malignant epidermal tumors.

## Background

The most common forms of human epidermal tumors include seborrheic keratosis, precancerous lesions such as Bowen's disease or bowenoid papulosis, and basal or squamous cell carcinoma. Seborrheic keratosis is a benign form of hyperplasia involving epidermal basaloid cells and keratinocytes. Bowen's disease is very similar to squamous cell carcinoma. Atypical squamous cells proliferate throughout the entire thickness of the epidermis without invading the dermis. Bowenoid papulosis has a histological resemblance to Bowen's disease. In this condition atypical keratinocytes are seen at all levels of the epidermis, but the cells are less atypical than those seen in Bowen's disease. Both conditions have the potential to progress into squamous cell carcinoma.

Basal cell carcinoma is a slow-growing, locally invasive malignant skin tumor with low metastatic potential. It begins in the deep basal cell layer of the epidermis and is characterized by cancerous nests of basaloid cells that extend into the dermis. Squamous cell carcinoma begins as a locally invasive malignant skin tumor. Cancerous nests of atypical squamous cells arise from different layers of the epidermis and extend irregularly into the dermis. Both the malignant and metastatic potential of squamous cell carcinoma are relatively high.

The mechanism of tumorigenesis and progression has been shown to be related to the local inflammatory reactions, especially chronic persistent inflammation [1-3]. These tumors are not generally associated with pathogenic infection, suggesting that endogenous factors trigger local inflammation via the release of damage associated molecule pattern (DAMP) molecules, containing high mobility group protein box 1 (HMGB1) and heat shock protein 70 (HSP70) [4,5].

HMGB1 is a DNA binding protein located in nucleus, which is released into the extracellular fluid in the presence of inflammation and cell necrosis [6,7]. Extracellular HMGB1 is, therefore, considered to be an important proinflammatory cytokine which acts by binding to toll-like receptor 4 (TLR4) receptors [8-10]. TLR4 is controlled by pattern recognition receptors (PRR) which are able to distinguish between pathogens and DAMP. It is predominantly expressed in antigen-presenting cells (APC) including dendritic cells (DC), macrophages and also in tumor cells.

Extracellular HMGB1 binds to TLR4 and causes myeloid differentiation primary response gene 88 (MyD88) to activate nuclear factor kappa-light-chain-enhancer of activated B cells (NF-κB) [11]. Activated NF-κB is transported to the nucleus from the cytoplasm, where it induces expression of inflammatory factors and promotes cell proliferation and anti-apoptosis. In this way it plays an important role in tumor genesis and progression [12].

It has been recognized that HMGB1 plays an important role in autoimmunity disease and cancers [13], and HMGB1, TLR4 and NF-κB have all been shown to participate in the progression and metastasis of malignant tumors [14,15]. However, the effects of these mediators in seborrheic keratosis, precancerous lesions, basal cell carcinoma and squamous cell carcinoma have not been clarified. We, therefore, investigated their involvement in the different types of skin tumors primarily by exploring the relationship between HMGB1-TLR4 pathway related inflammation and tumor development.

## Methods

### Subjects and specimens

Human epidermal tumor specimens were obtained from 28 patients with seborrheic keratosis, 12 patient with precancerous lesions, 21 patients with basal cell carcinoma, and 28 patients with squamous cell carcinoma. Tumor diagnosis was based on clinical and histopathological criteria. A 3-week 'washout' period from the effects of radiotherapy or immunotherapy was implemented before specimen collection. Participants with immune deficiency diseases were excluded from the study.

Normal skin specimens were obtained from seven healthy subjects undergoing surgical circumcision or orthopaedic procedures. Pathological examination of each specimen was performed using hematoxylin-eosin stained sections.

The study was performed in accordance with the Declaration of Helsinki 1964 and its later amendments. The protocol was approved by the Clinic Research Ethics Board of Tongji Medical College. All participants provided a written informed consent prior to inclusion in the study.

### Antibodies and reagents

The antibodies used for immunohistochemistry and western blot analysis included anti-HMGB1 (EPITOMICS, 2600–1), anti-TLR4 (Abcam, ab22048), anti-NF-κB p65 (Santa Cruz, SC-7151), REAL™EnVision Detection Kit (Dako), and anti-HSP70 (Abcam, ab47455).

### Immunohistochemistry

EnVision was used to detect expression of HMGB1, TLR4 and NF-κB p65 in human epidermal tumor and normal skin specimens. HSP70 was also tested in both tumor and normal specimens.

The 96 tissue specimens were routinely fixed in formalin and embedded in paraffin. Sections 4 μm thick were cut from paraffin-embedded tissue blocks and mounted on silanized slides. After de-waxing and rehydration, the sections were antigen retrieved with ethylenediamine tetraacetic acid or citric acid, incubated with 3% H_2_O_2_ for 10 min and blocked with 5% BSA for 20 min. The specimens were then incubated with the primary antibodies (anti-HMGB1 1:800, anti-TLR4 1:200, anti- NF-κB p65 1:200, anti-HSP70 1:100) for 24 h at 4°C. Next the secondary antibodies was added (ChemMateTMEnVision +/HRP) and the specimens were incubated for 45 min, followed by the addition of 50 to 100 μL of diaminobenzidine (DAB).

Dehydration, transparence, mounting and microscopic examination were prepared using routine procedures. The specimens were photographed with a Nikon Eclipse Ti-SR microscope equipped with a Nikon DS-U3 digital camera. Negative controls were obtained by omitting the primary antibodies.

The immunohistochemistry grading of the nucleus, cytoplasm, cell membrane, cell or intercellular space was undertaken semi-quantitatively by two blinded pathologists. The average scores were used for analysis. The scoring system was as follows: 0 = no staining, 1 = light brown yellow, 2 = brown and 3 = dark brown staining. Ten fields were counted on each slide at 400 x magnification. The average positive expression on each slide was scored as: 1 = <25%, 2 = 25 to <50%, 3 = 50 to <75% and 4= > 75%. The product of the positive expression percentage and degree of staining scores for each slide provided a final score where: 0 to 1 point was negative (-), 2 to 3 points was weakly positive (+), 4 to 6 points was moderately positive (+ +), and >6 points was strongly positive (+ + +).

### Western blot analysis

Western blot analysis was used to detect the expression of NF-κB p65 in epithelial cell nuclei in human epidermal tumor and normal tissues. Dermis and subcutaneous tissues were removed from the specimens and the epidermis was cut into small pieces for western blot analysis. Epithelial nuclear proteins were prepared from the tissues using a cytoplasmic/nuclear extraction kit. Equal amounts of cytoplasmic and nuclear extracts were subjected to 10% sodium dodecyl sulfate polyacrylamide gel electrophoresis (SDS-PAGE) and transferred to nitrocellulose membranes. The membranes were blocked overnight at 4°C in buffer containing 5% non-fat dried milk in phosphate buffered saline (PBS) and 0.1% Tween-20. The membranes were then blotted for 2 h at room temperature with the primary antibody, anti-NF-κB p65, diluted at 1:500. The membrane-bound antibodies were labeled using horseradish peroxidase-conjugated (HRP) anti-IgG diluted at 1:3000. Histone H3 was used as a loading control. An enhanced chemiluminescence system (Pierce) was used for detection.

### Statistical analysis

Statistical analysis was undertaken using Stata version 11.0 software. Data were expressed as the means and standard errors (±SEM). Between-group differences were analyzed by one-way analysis of variance (ANOVA) followed by Bonferroni method for normally distributed datasets. The Kruskal-Wallis test followed by Nemenyi Multiple Comparison test was used for skewed datasets. The correlation analysis was performed using Spearman’s correlation test. Values of P <0.05 were considered statistically significant, and P <0.01 were considered extremely statistically significant.

## Results

### Expression of HMGB1 in human epidermal tumors

**Figure 1:**
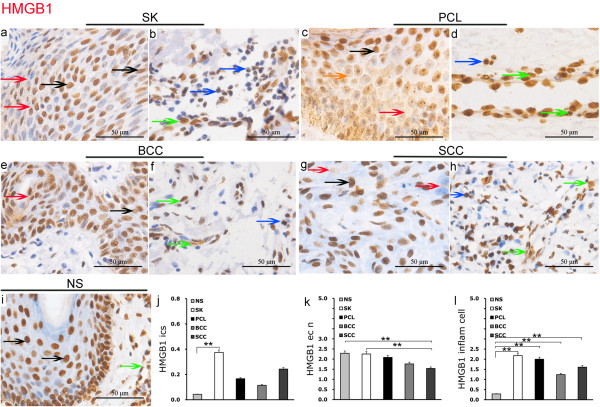
**Expression of HMGB1 in epidermal tumors and normal skin by IHC EnVision (magnification × 400).** (**a**) to (**i**). Positive expression of HMGB1 was located in the nucleus, cytoplasm, cell, and (or) intercellular space after stimulation of inflammation or cell necrosis. The red arrow shows HMGB1 expression in the epithelial intercellular space, the black arrow shows positive HMGB1 expression in epithelial cell nuclei, the orange arrow shows HMGB1 expression in the epithelial cell cytoplasm, the blue arrow shows HMGB1 expression in an inflammatory cell, and the green arrow shows HMGB1 expression in a vascular endothelial cell. (**j**). **P < 0.01 of HMGB1 in epithelial intercellular spaces in SK as compared in NS. (**k**). **P < 0.01 of HMGB1 in epithelial cell nuclei in NS and SK as compared in SCC. (**l**). **P <0.01 as compared HMGB1 in inflammatory cells in NS. The error bars show the standard error of the mean (SEM).

In benign seborrheic keratosis (SK), HMGB1 exhibited diffuse strong positive expression in squamous epithelial nuclei with little evidence of positive focal expression in the cytoplasm. Extracellular HMGB1 was also extensively present in epithelial intercellular spaces. In both the nucleus and cytoplasm of inflammatory cells, there was strong positive diffusive expression of HMGB1. HMGB1 was also present in the nucleus and cytoplasm of vascular endothelial cells (Figure 1a and 1b).

In precancerous lesions (PCL), HMGB1 exhibited diffusive positive expression in the epithelial nuclei with focal expression in the cytoplasm. Scattered expression of HMGB1 was seen in the epithelial intercellular spaces of these cells. The nucleus and cytoplasm of associated inflammatory and vascular endothelial cells showed diffusive positive expression of HMGB1 (Figure 1c and 1d).

In low malignant basal cell carcinoma (BCC), there was diffuse moderate positive expression of HMGB1 in the cancerous epithelial nuclei, and the cytoplasm exhibited focal positive expression. Occasional sporadic expression of HMGB1 was seen in the intercellular spaces together with positive expression in the nucleus and cytoplasm of associated inflammatory and vascular endothelial cells (Figure 1e and 1f).

In highly malignant squamous cell carcinoma (SCC), there was relatively weak diffuse positive expression of HMGB1 in the cancerous epithelial nuclei, but minimal expression in the cytoplasm and scattered expression of HMGB1 in the epithelial intercellular spaces. There was positive expression of HMGB1 in associated inflammatory cells, both in the nucleus and cytoplasm, together with positive expression of HMGB1 in the nucleus and cytoplasm of vascular endothelial cells (Figure 1g and 1h).

Interestingly, HMGB1 exhibited strong positive diffuse expression in the nuclei of normal squamous epithelial cells and occasional positive focal expression in the cytoplasm. There was minimal HMGB1 expression in the intercellular spaces of the normal squamous epithelium and there were few inflammatory cells showing minimal evidence of nuclear or cytoplasmic expression of HMGB1. However, both the nucleus and cytoplasm of vascular endothelial cells in normal skin showed a strong positive expression of HMGB1 (Figure 1i).

Analysis of variance showed the expression of HMGB1 in epithelial intercellular spaces of benign seborrheic keratosis was significantly higher than in normal skin (P =0.0025), but there was no significant difference between seborrheic keratosis and highly malignant squamous cell carcinoma (Figure 1j). Expression of HMGB1 in the epithelial nuclei of highly malignant squamous cell carcinoma was significantly lower than in normal skin and in benign seborrheic keratosis (P = 0.003), but there was no significant difference between seborrheic keratosis, precancerous lesions, basal cell carcinoma and normal skin (Figure 1k).

Expression of HMGB1 in inflammatory cells of seborrheic keratosis, precancerous lesions, basal cell carcinoma and squamous cell carcinoma was significantly higher than in normal skin (P = 0.0024); HMGB1 expression in inflammatory cells of benign seborrheic keratosis increased non-signigicantly (Figure 1l).

### Expression of TLR4 in human epidermal tumors

**Figure 2:**
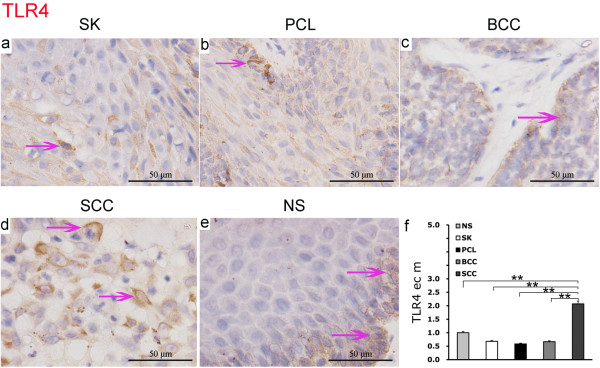
**Expression of TLR4 in epidermal tumors and normal skin by IHC EnVision (magnification × 400).** (**a**) to (**e**). Positive expression of TLR4 was located on the epithelial membrane (purple arrow). (**f**). **P <0.01 as compared TLR4 on epithelial cell membranes in SCC. The error bars represent the standard error of the mean (SEM).

In benign seborrheic keratosis (SK) and precancerous lesions (PCL), there were diffuse positive expression of TLR4 on epithelial cell membranes (Figure 2a and 2b). In basal cell carcinoma (BCC), TLR4 expression was seen on cell membranes of the cancerous epithelium (Figure 2c) and in highly malignant squamous cell carcinoma (SCC), there was a strong membrane positive expression of TLR4 on almost all of the cancerous epithelium (Figure 2d). In normal skin (NS), TLR4 expression was found with focal expression on the epithelial cell membranes (Figure 2e).

In squamous cell carcinoma, expression of TLR4 on epithelial cell membranes was significantly higher than in seborrheic keratosis, precancerous lesions, basal cell carcinoma and normal skin (P = 2.3e-5). There was no significant difference between TLR4 expression in seborrheic keratosis, precancerous lesions, basal cell carcinoma and normal skin (Figure 2f).

### Expression of NF-κB p65 in human epidermal tumors

**Figure 3:**
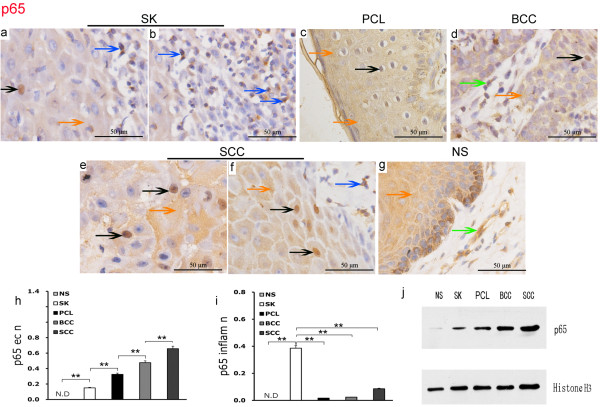
**Expression of p65 in epidermal tumors and normal skin by 96 IHC EnVision (magnification × 400).** (**a**) to (**g**). Positive expression of p65 was located in the cytoplasm, cell, and (or) nucleus after activation. The black arrow shows expression of p65 in the epithelial nucleus, the orange arrow shows p65 expression in the epithelial cytoplasm, the blue arrow shows p65 expression in an inflammatory cell and the green arrow shows p65 expression in a vascular endothelial cell. (**h**). **P < 0.01 as compared with p65 in the epithelial nuclei in different groups (**i**). **P < 0.01 as compared p65 in inflammatory cell nuclei in SK. The error bars represent the standard error of the mean (SEM). (**j**). Western blot detection of p65 expression in epithelial nuclei, which increased gradually from NS, SK, PCL, BCC, and to SCC. Histone H3 was used as a loading control.

In benign seborrheic keratosis (SK), p65 exhibited relatively weak expression in the epithelial nuclei but there was evidence of focal expression in the cytoplasm. In the associated inflammatory cells there was relatively strong p65 expression in the nucleus, and focal positive expression in the cytoplasm. In vascular endothelial cells there was weak expression of p65 in the nucleus and focal positive expression in the cytoplasm (Figure 3a and 3b).

There was weak expression of p65 in the nucleus and cytoplasm of precancerous lesion (PCL) epithelial cells. There was also weak p65 expression in the nucleus of associated inflammatory cells with focal positive expression in the cytoplasm. Expression of p65 expression was sporadic in the nucleus and cytoplasm of vascular endothelial cells (Figure 3c).

In malignant basal cell carcinoma (BCC), p65 was expressed in the epithelial nuclei and there was positive focal expression in the cytoplasm. In associated inflammatory cells, there was weak p65 expression in the nucleus and focal expression in the cytoplasm, and there was weak p65 expression in the nucleus and cytoplasm of vascular endothelial cells (Figure 3d).

Malignant squamous cell carcinoma (SCC) showed relatively high p65 expression in the nucleus and diffuse positive expression in the cytoplasm. p65 was also expressed in the nucleus of associated inflammatory cells, with sporadic positive expression in cytoplasm, together with evidence of expression in the nucleus and cytoplasm of associated vascular endothelial cells (Figure 3e and 3f). By contrast, in normal skin (NS), there was almost no p65 expression in the epithelial nuclei but focal positive expression was found in the epithelial cytoplasm. Minimal p65 expression was seen in inflammatory cells, and vascular endothelial cells in normal skin displayed occasional focal p65 expression in the cytoplasm with no nuclear expression (Figure 3g).

Analysis of variance indicated that expression of p65 in the epithelial nuclei of different epidermal tumors was higher than in normal skin. The level of p65 epithelial nuclear expression was increased progressively from normal skin, benign hyperplasia, precancerous lesions, low malignancy to high malignancy tumors (P = 0.0025; Figure 3h). The expression of p65 in inflammatory cell nuclei associated with benign seborrheic keratosis was significantly higher than in normal skin, precancerous lesions, basal cell carcinoma and squamous cell carcinoma (P =0.007). There was no significant difference between precancerous lesions, basal cell carcinoma and squamous cell carcinoma and normal skin (Figure 3i).

Western blot detection identified different levels of p65 expression in epithelial nuclei between normal skin and different tumors. There was almost no p65 expression in the epithelial nuclei of normal skin, whereas the epithelial nuclei of malignant basal cell carcinoma and malignant squamous cell carcinoma showed significant expression. In addition, p65 expression in squamous cell carcinoma was higher than in basal cell carcinoma, and p65 expression in epithelial cell nuclei of precancerous lesions was higher than in seborrheic keratosis (Figure 3j). Taken together, these findings suggest p65 expression in epithelial nuclei is upregulated with increased epithelial cell malignancy.

### Expression of HSP70 in human epidermal tumors

**Figure 4:**
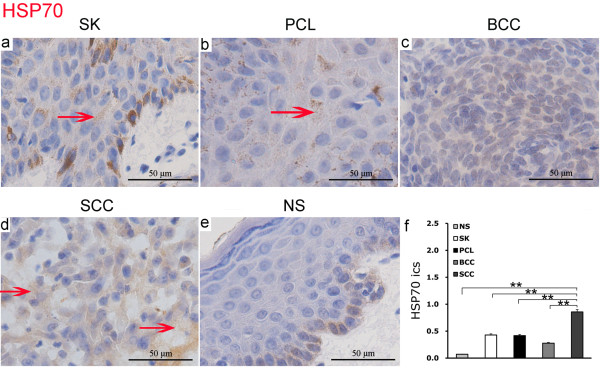
**Expression of HSP70 in epidermal tumors and normal skin by IHC EnVision (magnification × 400).** (**a**) to (**e**). Positive expression of HSP70 was located in the epithelial intercellular space as shown by the red arrows. (**f**). **P <0.01 as compared HSP70 in the epithelial intercellular spaces in SCC. The error bars represent the standard error of the mean (SEM).

Positive expression of HSP70 was found in the epithelial intercellular spaces in benign seborrheic keratosis (SK) and precancerous lesions (PCL) (Figure 4a and 4b). There was also evidence of HSP70 expression in basal cell carcinoma (BCC) (Figure 4c), with relatively strong positive expression in the epithelial intracellular spaces of highly malignant squamous cell carcinoma (SCC) (Figure 4d). Minimal HSP70 expression was found in the epithelial intercellular spaces of normal skin (NS) (Figure 4e).

Analysis of variance showed that HSP70 expression in epithelial intercellular spaces of squamous cell carcinoma was significantly higher than in normal skin, seborrheic keratosis, precancerous lesions and basal cell carcinoma (P = 0.0077). There was no significant difference in HSP70 expression between seborrheic keratosis, precancerous lesions, basal cell carcinoma and normal skin (Figure 4f).

### Correlation analysis

**Figure 5:**
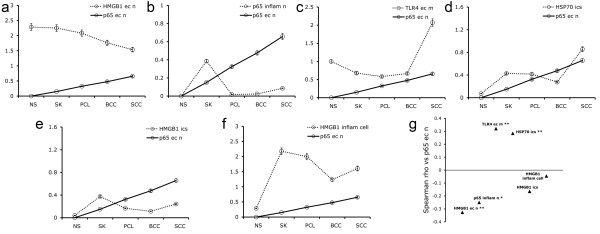
**Correlation analysis of IHC.** (**a**)-(**f**). The line-charts with SEM showing expression of p65 in epithelial cell nuclei and other mediators by Spearman's correlation analysis. (**g**). The correlation coefficients of Spearman rho as compared p65 in epithelial nuclei. (**a**) and (**g**). p65 in epithelial nuclei as compared HMGB1 in the epithelial cell nuclei ((r = -0.3264, **P <0.01). (**b**) and (**g**). p65 in epithelial nuclei as compared p65 in the inflammatory nuclei (r = -0.2496, *P <0.05). (**c**) and (**g**). p65 in epithelial nuclei as compared TLR4 on the epithelial cell membranes (r = 0.3212, **P <0.01). (**d**) and (**g**). p65 in epithelial nuclei as compared HSP70 in the epithelial intercellular spaces (r = 0.2844, **P <0.01). (**e**) and (**g**). p65 in epithelial nuclei as compared HMGB1 in the epithelial intercellular spaces (r = -0.1641, P > 0.05). (**f**) and (**g**). p65 in epithelial nuclei as compared HMGB1 in the inflammatory cells (r = -0.0452, P > 0.05).

Spearman's correlation analysis showed that the expression of p65 in epithelial nuclei of normal skin and different tumors was negatively correlated with HMGB1 levels in the epithelial cell nuclei (r = -0.3264, P = 0.0012; Figure 5a and 5g), was negatively correlated with p65 levels in the inflammatory cell nuclei (r = -0.2496, P = 0.0142; Figure 5b and 5g), was positively correlated with TLR4 levels on the epithelial cell membranes (r = 0.3212, P = 0.0014; Figure 5c and 5g), and was positively correlated with HSP70 in the epithelial intercellular spaces in normal skin and various tumor types (r = 0.2844, P = 0.005; Figure 5d and 5g).

In addition, p65 in epithelial nuclei was negatively correlated with HMGB1 in the epithelial intercellular spaces (r = -0.1641, P > 0.05; Figure 5e and 5g), and was negatively correlated with HMGB1 in the inflammatory cells in normal skin and various tumor types (r = -0.0452, P > 0.05; Figure 5f and 5g).

## Discussion

Human epidermal tumors predominantly include benign seborrheic keratosis, precancerous lesions Bowen's disease or bowenoid papulosis, together with malignant basal cell carcinoma and highly malignant squamous cell carcinoma. It has been affirmed that some forms of tumorigenesis are closely related with chronic inflammation. It has also been reported that chronic hepatitis B can induce hepatocellular carcinoma, and that chronic gastritis or gastric ulcer can induce gastric cancer [16]. However the role played by HMGB1-TLR4 related inflammation in the development of human epidermal tumors remains unknown.

HMGB1 was initially identified as a widely existing DNA binding protein, which changes the chromatin or DNA configuration and regulates the transcription complex formation [6]. HMGB1 is actively produced by macrophages and monocytes; it is passively released by damaged or necrotic cells and is thought to be involved in tumor cell invasion and metastasis [6,17]. Extracellular HMGB1 has been shown to act as a proinflammatory cytokine, which binds to TLR4, TLR2 or receptors for advanced glycation end-products (RAGE) [18-20]. It has also been reported that HMGB1 activates the MAPK-NF-κB pathway by interacting with RAGE, and that it plays an important role in inflammation [20-22].

Toll proteins, first found in Drosophila spp [23,24], are type I transmembrane proteins [25]. TLR4 is able to recognize and interact with HMGB1, HSP70 or lipopolysaccharide (LPS) to mediate signal transduction pathways, including MyD88-dependent and independent pathway [26,27]. NF-κB activation and cytokine production are both thought to be mediated by the MyD88-dependent pathway [28]. NF-κB is a widely expressed molecule with a wide range of biological functions including a role in regulating inflammation [29], cell differentiation, apoptosis and cell proliferation [30]. It has also been associated with tumorgenesis, cell invasion, metastasis and apoptosis [31-33]. NF-κB family members form two dimers with homologous or heterologous forms, the most common dimer being the combination of p50 and p65. NF-κB is formed by the heterologous dimerization of p50 and p65, and NF-κB p65 acts an important nucleus transcription factor [31,34].

NF-κB promotes malignancy through a number of mechanisms [35,36]. Activated NF-κB acts as an anti-apoptotic factor which induces or up-regulates anti-apoptotic genes and inhibits apoptosis. The activation of NF-κB causes cyclinD1 to promote tumor cell proliferation and independent division. NF-κB also activates the transcription and translation of a variety of genes that control tumor cell adhesion and angiogenesis. These include IL-8, tenascin C, cell adhesion molecule-1, and matrix metalloproteinase-3. Various studies have reported that NF-κB expression and activation are abnormal in breast, thyroid, colon, and stomach cancer, and in some other malignancies [15].

These findings prompted us investigate the diversity of expression and role played by HMGB1, TLR4 and p65 in epidermal tumors. We focused our attention on the role played by extracellular HMGB1 expression in epithelial intercellular spaces, TLR4 expression on epithelial cell membranes and p65 expression in epithelial nuclei. We selected various stages of the epidermal tumor tissues including seborrheic keratosis, squamous cell carcinoma in situ, basal cell carcinoma, and squamous cell carcinoma. The clinical pathological process associated with these conditions ranged from benign hyperplasia, to precancerous lesions, to low and high-grade malignancy. Normal skin specimens were used as controls.

Immunohistochemistry results showed that HMGB1 was differentially expressed in epithelial intercellular spaces, with seborrheic keratosis and squamous cell carcinoma showing higher expression than normal skin. This finding implies that intracellular HMGB1 is released from various epidermal tumors as a result of cell necrosis cells, enabling it to act as an extracellular mediator of local inflammation. However, HMGB1 expression in the intracellular space in highly malignant squamous cell carcinoma was lower than in seborrheic keratosis, whereas epithelial nuclear expression of p65, which indicative of NF-κB activation as well as inflammation responses, was higher in squamous cell carcinoma than in all other cell types (P <0.01). These findings suggest that HMGB1 maybe not be the principal mediator of inflammation in highly malignant skin tumors. Correlation analysis showed that expression of HMGB1 in epithelial intercellular spaces was negatively correlated with p65 in the epithelial nuclei but did not reach statistical significance, indicating that extracellular HMGB1 may not play a central role in highly malignant tumors. Furthermore, the expression of HMGB1 in epithelial nuclei in squamous cell carcinoma was significantly lower than in normal skin and benign seborrheic keratosis (P <0.01), suggesting that HMGB1 maybe not account for epidermal tumor progression. Instead, HMGB1 in the nucleus may contribute to the stabilization of DNA and chromosomes in epidermal tumors.

We also found that the membrane expression of TLR4 was higher in squamous cell carcinoma than normal skin and other tumors (P <0.01). TLR4 has been previously shown to interact with extracellular HMGB1 to activate NF-κB [11]. Taken together these findings suggest that TLR4 signaling pathways may act as mediators of increased inflammation in high malignancy epidermal tumors.

Immunohistochemistry also indicated different levels of expression of p65 in the epithelial nuclei of epidermal tumors. There was relatively low epithelial nuclear expression in benign seborrheic keratosis, with higher levels of expression in precancerous lesions and basal cell carcinoma, relatively strong expression in highly malignant squamous cell carcinoma. In contrast, there was almost no nuclear expression of p65 in normal skin. Western blot analysis showed similar results, indicating a tendency towards increased epithelial nuclear expression of p65 with increased levels of malignancy. The activation and nuclear translocation of NF-κB are both regulated by its inhibitory factor IκB. In the resting state, NF-κB dimer and IkB co-exist as a trimer which is concealed in the cytoplasm. This process explains why there was almost no squamous epithelial nuclear p65 expression in normal skin. We also demonstrated that the level of p65 epithelial nuclear expression increased progressively but significantly with tumor evolution from benign hyperplasia, to high level malignancy (P <0.01), suggesting the inflammation increases in parallel with tumor malignancy. This finding may be explained by NF-κB p65 activation, which induces the expression of inflammatory factors and promotes cell proliferation and anti-apoptosis.

The expression of NF-κB p65 in the nuclei of inflammatory cells associated with seborrheic keratosis was significantly higher than in other tissues (P <0.01). The expression of HMGB1 in inflammatory cells of seborrheic keratosis was also relatively strong. These findings suggest that inflammation associated with benign epidermal tumors may be mediated by inflammatory cells, and that inflammation of epidermal malignant tumors may not originate from inflammatory cells but from the malignant squamous epithelial cells themselves.

Correlation analysis showed that expression of p65 in epithelial nuclei was negatively correlated with HMGB1 expression in the epithelial nuclei in normal skin and in different tumors (r = -0.3264, P <0.01), further indicating that HMGB1 is not associated with epidermal tumor progression. We also showed that expression of p65 in epithelial nuclei was negatively correlated with p65 expression in inflammatory cell nuclei (r = -0.2496, P < 0.05), supporting the hypothesis that inflammation in epidermal benign tumors is mediated by inflammatory cells. In addition, the expression of p65 epithelial nuclei was positively correlated with TLR4 levels on the epithelium membrane (r = 0.3212, P <0.01), suggesting that p65 and TLR4 are both involved in epidermal malignant tumor genesis and progression. Thus, the TLR4-NF-κB p65 pathway appears to play a vital role in the development of malignancy.

However, there was no evidence that the same pathway was so intimately involved in highly malignant squamous cell carcinoma as expression of HMGB1 in the epithelial intercellular spaces was not higher than in other tumor types. It is possible, therefore, that other ligands engaging TLR4 such as HSP70 may also be involved. HSP70 is continually expressed in all living organisms and forms a significant part of the cellular machinery for protein folding, for protecting cells from stress [37]. Extracellular HSP70 has been shown to interact with TLR4, activate NF-κB signals and mediate inflammatory reactions [38-40].

Expression of HSP70 in the epithelial intercellular space of squamous cell carcinoma was significantly higher than in normal skin, seborrheic keratosis, precancerous lesions and basal cell carcinoma (P <0.01), and was positively correlated with the expression of p65 in epithelial nuclei (r = 0.2844, P <0.01), indicating that HSP70 may be another mediator of local inflammation in high malignancy epidermal tumors. These results also explain why HMGB1 expression in epithelial intercellular spaces of high malignancy squamous cell carcinoma was lower than seen with seborrheic keratosis.

## Conclusion

In conclusion, we elucidated that HMGB1 may be one of mediators resulting in the development of inflammation in epidermal tumors, but that it did not play a central role in highly malignant epidermal tumors. In these tumors the TLR4 signaling pathway appeared to be primarily involved in inducing inflammation. Inflammation intensified in parallel with the evolution of tumor malignancy and may also involve HSP70. We also showed that NF-κB p65 and TLR4 might play a significant role in the high malignancy epidermal tumors, and combined detection of TLR4 on epithelial cell membranes and p65 in epithelial cell nuclei may be useful for the diagnosis of the epidermal malignant tumors.

Furthermore, expression of HMGB1, TLR4, p65 and HSP70 in epidermal tumors and normal skin with more fields of vision could be seen in Additional file 1: Figure S1 of appendant, Additional file 2: Figure S2 of appendant, Additional file 3: Figure S3 of appendant and Additional file 4: Figure S4 of appendant, respectively.

## Abbreviations

BCC: Basal cell carcinoma; DAMP: Damage associated molecule prttern; HMGB1: High mobility group protein box 1; HSP: Heat shock protein; IHC: Immunohistochemistry; NF-κB: Nuclear factor-κB; PCL: Precancerous lesions; SCC: Squamous cell carcinoma; SK: Seborrheic keratosis; TLR4: Toll like receptor 4.

## Competing interests

The authors declare that they have no competing interests.

## Authors’ contributions

FLG and HW conceived of the study. HW carried out the experiments and drafted the manuscript. YHD and YYX carried out the immunohistochemistry analysis. HBL performed the statistical analysis. All authors read and approved the final manuscript.

## Pre-publication history

The pre-publication history for this paper can be accessed here:

http://www.biomedcentral.com/1471-2407/13/311/prepub

## Supplementary Material

Additional file 1: Figure S1 of Appendant Expression of HMGB1 in epidermal tumors and normal skin by IHC EnVision. (magnification × 400, larger field). Click here for file

Additional file 2: Figure S2 of Appendant Expression of TLR4 in epidermal tumors and normal skin by IHC EnVision. (magnification × 400, larger field). Click here for file

Additional file 3: Figure S3 of Appendant Expression of p65 in epidermal tumors and normal skin by IHC EnVision. (magnification × 400, larger field). Click here for file

Additional file 4: Figure S4 of Appendant Expression of HSP70 in epidermal tumors and normal skin by IHC EnVision. (magnification × 400, larger field). Click here for file

## References

[B1] Barta P Van Pelt C Men T Dickey BF Lotan R Moghaddam SJ Enhancement of lung tumorigenesis in a Gprc5a knockout mouse by chronic extrinsic airway inflammation Mol Cancer 2012 11 4 10.1186/1476-4598-11-4 22239913 PMC3281775

[B2] Kyewski B Romero P Chronic inflammation is regarded as a strong promoter of tumorigenesis Int J Cancer 2010 127 4 747 20564540 10.1002/ijc.25487

[B3] Carothers AM Davids JS Damas BC Bertagnolli MM Persistent cyclooxygenase-2 inhibition downregulates NF-{kappa}B, resulting in chronic intestinal inflammation in the min/+ mouse model of colon tumorigenesis Cancer Res 2010 70 11 4433 4442 10.1158/0008-5472.CAN-09-4289 20484034 PMC3242378

[B4] Jube S Rivera Z Bianchi ME Powers A Wang E Pagano IS Pass HI Gaudino G Carbone M Yang H Cancer cell secretion of the DAMP protein HMGB1 supports progression in malignant mesothelioma Cancer Res 2012 72 13 3290 3301 10.1158/0008-5472.CAN-11-3481 22552293 PMC3389268

[B5] Bianchi ME DAMPs, PAMPs and alarmins: all we need to know about danger J Leukoc Biol 2007 81 1 1 5 17032697 10.1189/jlb.0306164

[B6] Sims GP Rowe DC Rietdijk ST Herbst R Coyle AJ HMGB1 And RAGE in inflammation and cancer Annu Rev Immunol 2010 28 367 388 10.1146/annurev.immunol.021908.132603 20192808

[B7] Rovere-Querini P Capobianco A Scaffidi P Valentinis B Catalanotti F Giazzon M Dumitriu IE Muller S Iannacone M Traversari C HMGB1 Is an endogenous immune adjuvant released by necrotic cells EMBO Rep 2004 5 8 825 830 10.1038/sj.embor.7400205 15272298 PMC1299116

[B8] Dai S Sodhi C Cetin S Richardson W Branca M Neal MD Prindle T Ma C Shapiro RA Li B Extracellular high mobility group box-1 (HMGB1) inhibits enterocyte migration via activation of toll-like receptor-4 and increased cell-matrix adhesiveness J Biol Chem 2010 285 7 4995 5002 10.1074/jbc.M109.067454 20007974 PMC2836103

[B9] Akaike H Kono K Sugai H Takahashi A Mimura K Kawaguchi Y Fujii H Expression of high mobility group box chromosomal protein-1 (HMGB-1) in gastric cancer Anticancer Res 2007 27 1A 449 457 17352266

[B10] Ellerman JE Brown CK De Vera M Zeh HJ Billiar T Rubartelli A Lotze MT Masquerader: high mobility group box-1 and cancer Clin Cancer Res 2007 13 10 2836 2848 10.1158/1078-0432.CCR-06-1953 17504981

[B11] Tadie JM Bae HB Deshane JS Bell CP Lazarowski ER Chaplin DD Thannickal VJ Abraham E Zmijewski JW TLR4 Engagement inhibits AMPK activation through a HMGB1 dependent mechanism Mol Med 2012 9 18 659 668 22396017 10.2119/molmed.2011.00401 PMC3388138

[B12] Naugler WE Karin M NF-kappaB and cancer-identifying targets and mechanisms Curr Opin Genet Dev 2008 18 1 19 26 10.1016/j.gde.2008.01.020 18440219 PMC2587362

[B13] Tang D Kang R Zeh HJ 3rd Lotze MT High-mobility group box 1 and cancer Biochim Biophys Acta 2010 1799 1–2 131 140 20123075 10.1016/j.bbagrm.2009.11.014 PMC2818552

[B14] Liu PL Tsai JR Hwang JJ Chou SH Cheng YJ Lin FY Chen YL Hung CY Chen WC Chen YH High-mobility group box 1-mediated matrix metalloproteinase-9 expression in non-small cell lung cancer contributes to tumor cell invasiveness Am J Respir Cell Mol Biol 2010 43 5 530 538 10.1165/rcmb.2009-0269OC 19933377

[B15] Pacifico F Leonardi A NF-kappaB in solid tumors Biochem Pharmacol 2006 72 9 1142 1152 10.1016/j.bcp.2006.07.032 16956585

[B16] Peek RM Jr Fiske C Wilson KT Role of innate immunity in helicobacter pylori-induced gastric malignancy Physiol Rev 2010 90 3 831 858 10.1152/physrev.00039.2009 20664074 PMC2990353

[B17] Ito Y Bhawal UK Sasahira T Toyama T Sato T Matsuda D Nishikiori H Kobayashi M Sugiyama M Hamada N Involvement of HMGB1 and RAGE in IL-1beta-induced gingival inflammation Arch Oral Biol 2012 57 1 73 80 10.1016/j.archoralbio.2011.08.001 21861984

[B18] Agresti A Lupo R Bianchi ME Muller S HMGB1 Interacts differentially with members of the Rel family of transcription factors Biochem Biophys Res Commun 2003 302 2 421 426 10.1016/S0006-291X(03)00184-0 12604365

[B19] Kim SW Lim CM Kim JB Shin JH Lee S Lee M Lee JK Extracellular HMGB1 released by NMDA treatment confers neuronal apoptosis via RAGE-p38 MAPK/ERK signaling pathway Neurotox Res 2011 20 2 159 169 10.1007/s12640-010-9231-x 21116767

[B20] Nogueira-Machado JA Volpe CM Veloso CA Chaves MM HMGB1, TLR and RAGE: a functional tripod that leads to diabetic inflammation Expert Opin Ther Targets 2011 15 8 1023 1035 10.1517/14728222.2011.575360 21585289

[B21] Andersson U Rauvala H Introduction: HMGB1 in inflammation and innate immunity J Intern Med 2011 270 4 296 300 10.1111/j.1365-2796.2011.02430.x 21793949

[B22] Vitali R Stronati L Negroni A Di Nardo G Pierdomenico M Del Giudice E Rossi P Cucchiara S Fecal HMGB1 is a novel marker of intestinal mucosal inflammation in pediatric inflammatory bowel disease Am J Gastroenterol 2011 106 11 2029 2040 10.1038/ajg.2011.231 21788990

[B23] Hashimoto C Hudson KL Anderson KV The toll gene of drosophila, required for dorsal-ventral embryonic polarity, appears to encode a transmembrane protein Cell 1988 52 2 269 279 10.1016/0092-8674(88)90516-8 2449285

[B24] Wu B Huan T Gong J Zhou P Bai Z Domain combination of the vertebrate-like TLR gene family: implications for their origin and evolution J Genet 2011 90 3 401 408 10.1007/s12041-011-0097-3 22227927

[B25] Cook DN Pisetsky DS Schwartz DA Toll-like receptors in the pathogenesis of human disease Nat Immunol 2004 5 10 975 979 10.1038/ni1116 15454920

[B26] Pandey SC TLR4-MyD88 Signalling: a molecular target for alcohol actions Br J Pharmacol 2012 165 5 1316 1318 10.1111/j.1476-5381.2011.01695.x 21955082 PMC3372717

[B27] Bauerfeld CP Rastogi R Pirockinaite G Lee I Huttemann M Monks B Birnbaum MJ Franchi L Nunez G Samavati L TLR4-Mediated AKT activation is MyD88/TRIF dependent and critical for induction of oxidative phosphorylation and mitochondrial transcription factor a in murine macrophages J Immunol 2012 188 6 2847 2857 10.4049/jimmunol.1102157 22312125 PMC3294201

[B28] Hirano H Yoshioka T Yunoue S Fujio S Yonezawa H Niiro T Habu M Oyoshi T Sugata S Kamezawa T TLR4, IL-6, IL-18, MyD88 and HMGB1 are highly expressed in intracranial inflammatory lesions and the IgG4/IgG ratio correlates with TLR4 and IL-6 Neuropathology 2012 32 6 628 637 10.1111/j.1440-1789.2012.01310.x 22414145

[B29] Ghosh S Hayden MS New regulators of NF-kappaB in inflammation Nat Rev Immunol 2008 8 11 837 848 10.1038/nri2423 18927578

[B30] Karin M Greten FR NF-kappaB: linking inflammation and immunity to cancer development and progression Nat Rev Immunol 2005 5 10 749 759 10.1038/nri1703 16175180

[B31] Oeckinghaus A Hayden MS Ghosh S Crosstalk in NF-kappaB signaling pathways Nat Immunol 2011 12 8 695 708 10.1038/ni.2065 21772278

[B32] Siggers T Chang AB Teixeira A Wong D Williams KJ Ahmed B Ragoussis J Udalova IA Smale ST Bulyk ML Principles of dimer-specific gene regulation revealed by a comprehensive characterization of NF-kappaB family DNA binding Nat Immunol 2012 13 1 95 102 22101729 10.1038/ni.2151 PMC3242931

[B33] Espinosa L Bigas A Mulero MC Alternative nuclear functions for NF-kappaB family members Am J Cancer Res 2011 1 4 446 459 21984965 PMC3186045

[B34] Yadav VR Prasad S Gupta SC Sung B Phatak SS Zhang S Aggarwal BB 3-Formylchromone interacts with cysteine 38 in p65 protein and with cysteine 179 in IkappaBalpha kinase, leading to down-regulation of nuclear factor-kappaB (NF-kappaB)-regulated gene products and sensitization of tumor cells J Biol Chem 2012 287 1 245 256 10.1074/jbc.M111.274613 22065587 PMC3249075

[B35] Baeuerle PA Baltimore D NF-kappa B: ten years after Cell 1996 87 1 13 20 10.1016/S0092-8674(00)81318-5 8858144

[B36] Sen R Baltimore D Multiple nuclear factors interact with the immunoglobulin enhancer sequences Cell 1986 46 5 705 716 10.1016/0092-8674(86)90346-6 3091258

[B37] Ohtsuka K Hata M Molecular chaperone function of mammalian Hsp70 and Hsp40–a review Int J Hyperthermia 2000 16 3 231 245 10.1080/026567300285259 10830586

[B38] Tsan MF Gao B Heat shock proteins and immune system J Leukoc Biol 2009 85 6 905 910 10.1189/jlb.0109005 19276179

[B39] Wallin RP Lundqvist A More SH Von Bonin A Kiessling R Ljunggren HG Heat-shock proteins as activators of the innate immune system Trends Immunol 2002 23 3 130 135 10.1016/S1471-4906(01)02168-8 11864840

[B40] Krause M Rodrigues-Krause Jda C Extracellular heat shock proteins (eHSP70) in exercise: possible targets outside the immune system and their role for neurodegenerative disorders treatment Med Hypotheses 2011 76 2 286 290 10.1016/j.mehy.2010.10.025 21071151

