# Supplementary figures and images for: Expression and significance of HMGB1, TLR4 and NF-κB p65 in human epidermal tumors
Source: BMC Cancer. 2013 Jun 26;13:311. doi: 10.1186/1471-2407-13-311 (PMC3697986; doi:10.1186/1471-2407-13-311)

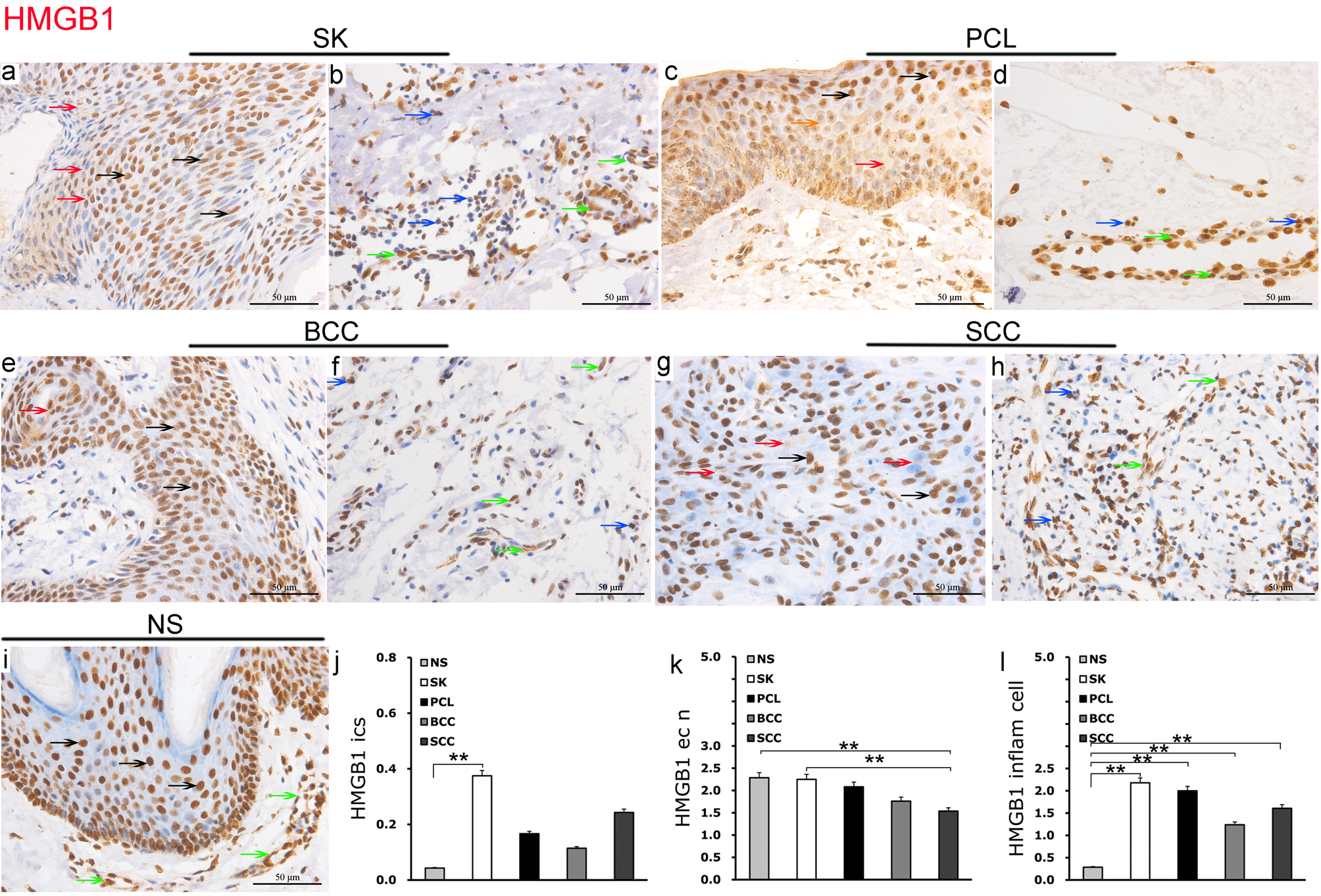

Supplement: Additional file 1: Figure S1 of Appendant — Expression of HMGB1 in epidermal tumors and normal skin by IHC EnVision. (magnification × 400, larger field). [file 1471-2407-13-311-S1.tiff]

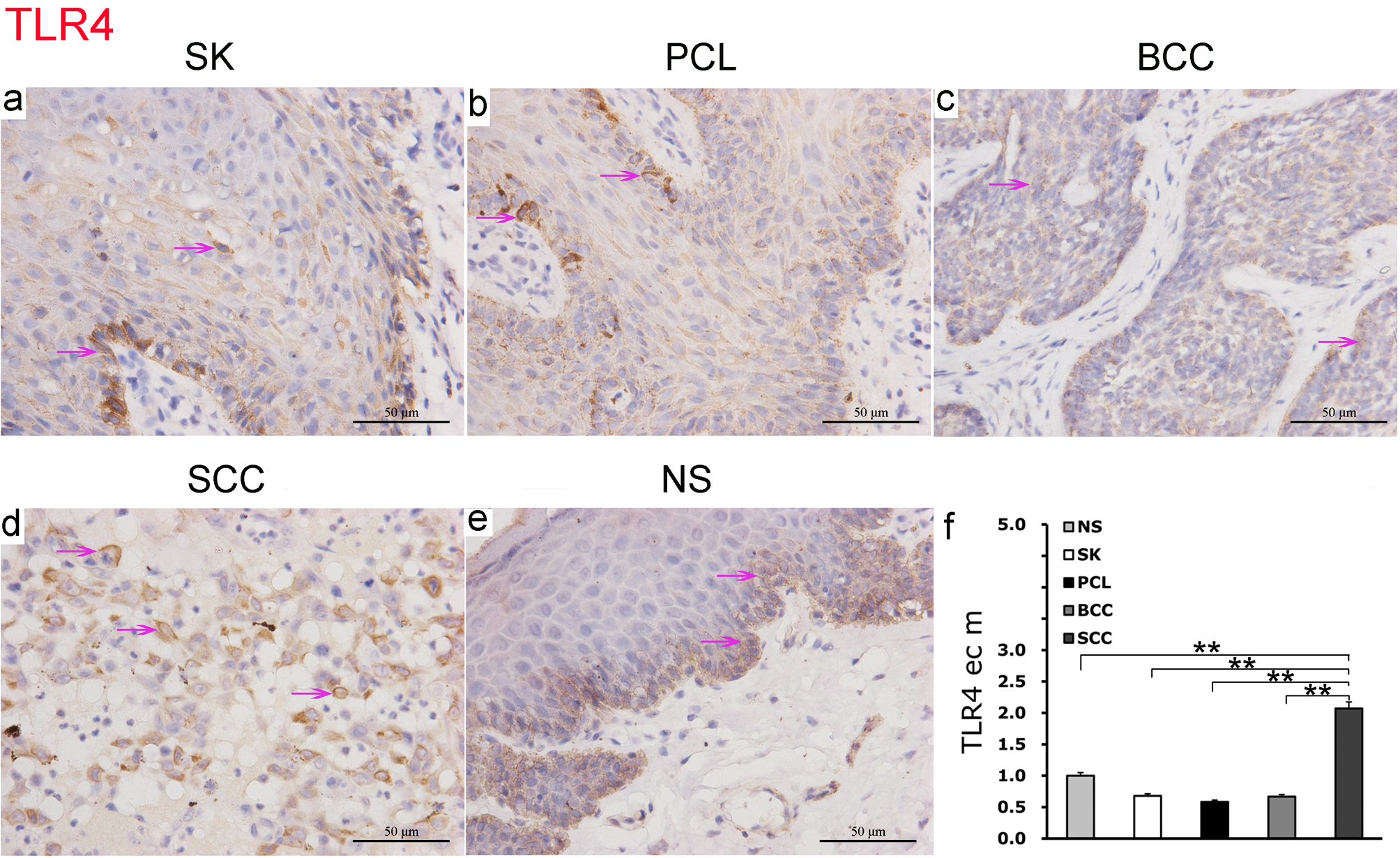

Supplement: Additional file 2: Figure S2 of Appendant — Expression of TLR4 in epidermal tumors and normal skin by IHC EnVision. (magnification × 400, larger field). [file 1471-2407-13-311-S2.tiff]

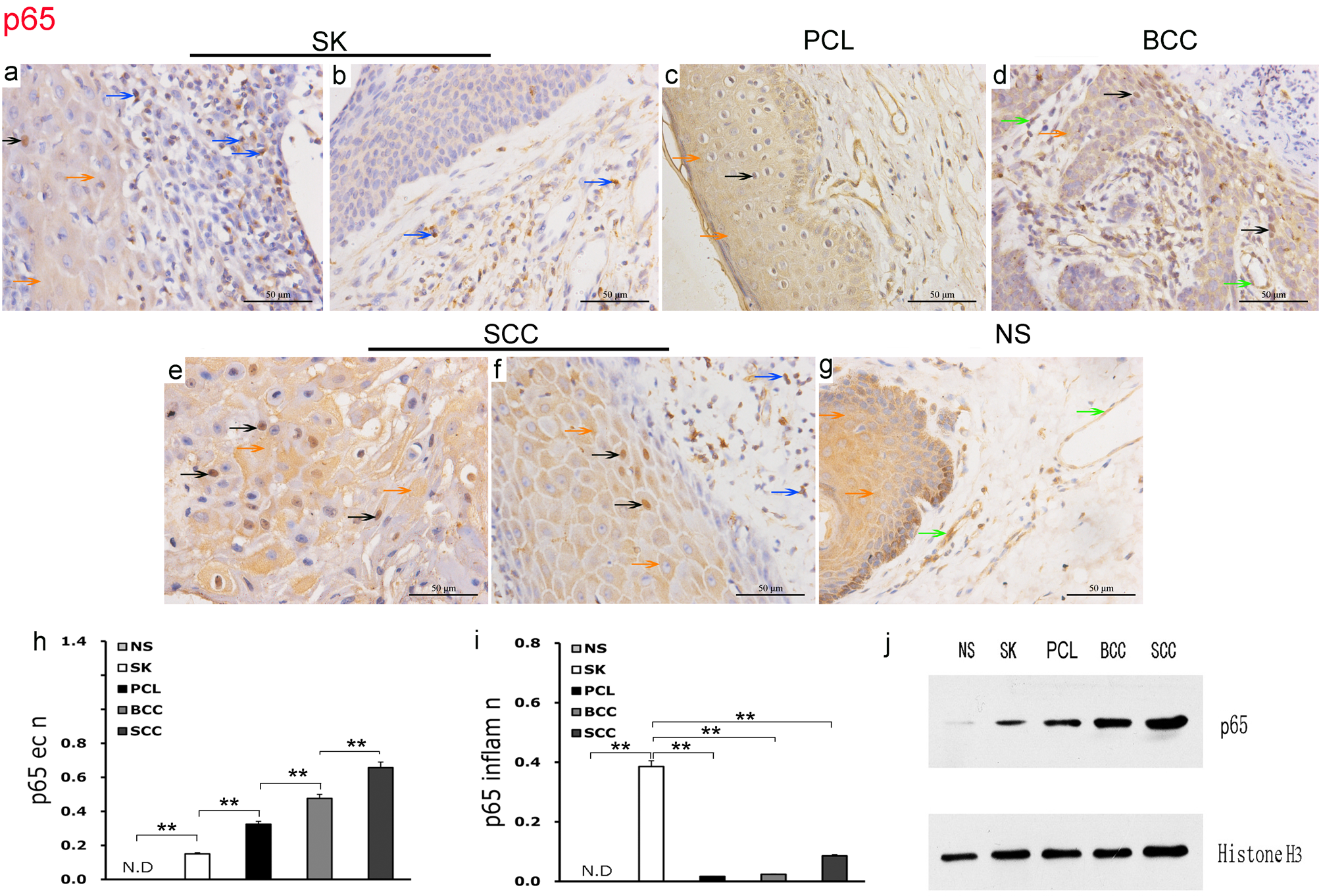

Supplement: Additional file 3: Figure S3 of Appendant — Expression of p65 in epidermal tumors and normal skin by IHC EnVision. (magnification × 400, larger field). [file 1471-2407-13-311-S3.tiff]

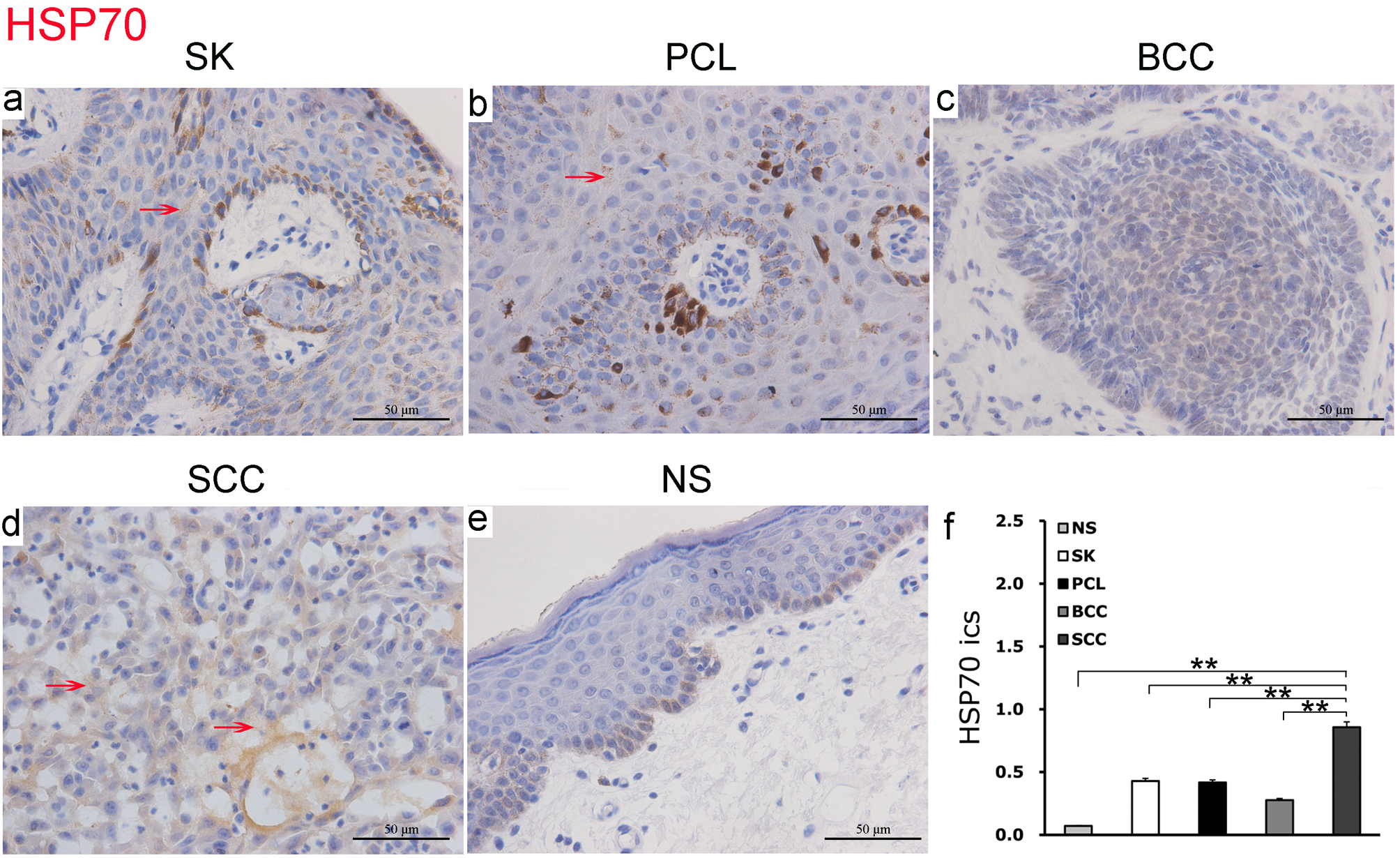

Supplement: Additional file 4: Figure S4 of Appendant — Expression of HSP70 in epidermal tumors and normal skin by IHC EnVision. (magnification × 400, larger field). [file 1471-2407-13-311-S4.tiff]
